# Adult children's unemployment and parental mental health in India: Social and economic heterogeneity

**DOI:** 10.1016/j.ssmph.2026.101905

**Published:** 2026-02-26

**Authors:** Rishabh Tyagi, Anna Baranowska-Rataj, Alexi Gugushvili

**Affiliations:** a Max Planck Institute for Demographic Research, Rostock, Germany; b Center for Fertility and Health, Norwegian Institute of Public Health, Norway; c Department of Sociology and Human Geography, University of Oslo, Norway; d Center for Demographic and Ageing Research, Umeå University, Sweden; e Centre for Demographic Studies (CED), The Autonomous University of Barcelona, Spain

**Keywords:** Parental mental health, Adult Children's unemployment, Social capital, Income inequality, India

## Abstract

This study explores the relationship between adult children's unemployment and parental mental health. Given India's large inequalities in social capital and income, we examine the heterogeneous effects of these factors on the relationship. We utilise data from the Longitudinal Ageing Survey of India, which includes 73,396 individuals aged 45 and above. We analyse the relationship between the exposure to the unemployment of adult children and the parental risk of depression using the CES-D score, with respondents reporting four or more symptoms out of 10 considered at risk of being “depressed”. We employ inverse probability weighting based on a logistic regression model to form a pseudo-control group, accounting for confounding demographic and socioeconomic characteristics. Our findings show a 3.14 percentage points (ppts) increase in absolute terms (and a 12.48% relative increase) in the probability of parental depression risk associated with adult children's unemployment. There are no significant differences between fathers and mothers in the increased risk of depression, but for the unemployment of the firstborn son, there is a significantly higher association of increased risk of depression than for the unemployment of the firstborn daughter. Heterogeneity analyses reveal that among older adults with high social participation, there is no significant increase in their risk of depression associated with their children's unemployment. Similarly, among older adults residing in low and medium-income inequality states, the negative consequences of their children's unemployment are weaker. Overall, we conclude that while adult children's unemployment is associated with an increased risk of parental depression, higher social participation and residing in low or medium-income inequality states might have protective effects on older adults' mental health following their children's unemployment. Governments may consider expanding labour market policies that support youth labour market entry as a means to improve not only the employability of younger individuals, but also the well-being of older generations.

## Highlights

• Adult Children's unemployment is associated with parents' increased depression risk.
• Firstborn sons' unemployment is strongly associated with parents' depression risk.
• Parents with High Social participation have no increased risk of depression.
• Parents in low and medium-income inequality states have a lower risk of depression.

## 1 Introduction

Parents with well-educated children tend to be more satisfied with life and to have healthier and longer lives than parents with children who are not well educated (Mustafa and Shekhar, 2024; Sabater, Graham and Marshall, 2020; Smith-Greenaway, Brauner-Otto and Axinn, 2018). A small but growing body of evidence suggests that how adult children fare in their working lives is a plausible mechanism behind these intergenerational cross-over effects. This is theoretically well-explained by the “linked lives” principle of life course research proposed by Elder Jr (1998), which states that human lives are lived interdependently within families, and the misfortunes of individual family members are shared through relationships. Thus, we can expect that exposure to their children's unemployment will affect parents' mental health. Offspring unemployment has been shown to negatively affect parents' psychological well-being and mental health in the UK (Albertini & Piccitto, 2023). The latter study also found that their children's transition to unemployment has a negative effect on mothers' mental well-being, whereas changes in fathers' distress levels are non-significant following their children's unemployment. Bister et al. (2025) additionally revealed that the strength of these associations varies across welfare state regimes and macro-economic contexts, and thus called for a more detailed examination of the factors that protect parents' health.

The life course literature situates individual health outcomes within broader social and institutional contexts (Hayward & Sheehan, 2016) and posits that social environments and institutions represent powerful forces that shape both exposures and responses to adverse experiences such as unemployment. Hence, while the “linked lives” perspective provides strong arguments for a negative relationship between adult children's adverse labour market experiences and parental mental health, this relationship is unlikely to be universal across all families and contexts (Bister et al., 2025). Recent studies have shown that adverse economic experiences within families may have quite different consequences depending on a broad range of individual and contextual factors (Aquino et al., 2022; Torche et al., 2024).

The literature on the heterogeneous effects of adverse events has focused on events that happen to parents, and on how national or sub-national policies can have a potentially protective effect on children (see, e.g., Baranowska-Rataj et al., 2024; Hansen & Stutzer, 2022). However, we argue that heterogeneous effects can also be expected when studying the reverse exposures, i.e., the influence of adult children's unemployment on parental mental health. We propose a novel framework to explain how high levels of social participation and low levels of income inequality may constitute contextual factors that mitigate the otherwise negative association between adult children's unemployment and parental well-being. Our selection of the contextual factors is based on the literature pointing to the key mechanisms behind the negative impact of adverse life events in the younger generation on the well-being of the older generation, specifically, social relations strain and financial strain. We argue that as long as the negative associations between adult children's unemployment and parental mental health are driven by relational and financial strains, then these associations should be weaker in contexts of high social participation and low levels of income inequality.

This study makes three important contributions to the literature. First, our research adds to the growing body of research on the heterogeneous effects of adverse experiences in human lives. Specifically, it expands the knowledge of factors that shield older adults' mental health when they are exposed to adverse economic circumstances among their adult children. Second, we advance the knowledge of health inequalities in India, the most populous country in the world. Previous research on the links between adult children's unemployment and parental mental health has been restricted to European societies (Albertini & Piccitto, 2023; Bister et al., 2025) and thus has not taken into account the experiences of countries in the Global South. India is a highly relevant context for the present study, given the prevalence of strong familial ties in the country's culture and the intergenerational financial transfers from adult children to their parents. India also exhibits vast heterogeneities in social participation and income at the state level (Chandrasekhar et al., 2021), which we expect to moderate the relationship between adult children's unemployment and their parents' risk of depression. Third, our study adds to the literature on the role of social capital and income inequalities in population health. Although a significant body of research has documented the positive association between social participation and income equality on population health (Moore & Kawachi, 2017; Pickett & Wilkinson, 2015; Villalonga-Olives & Kawachi, 2017), there is less research examining the same links at the individual level. Our study investigates not just whether social participation and income inequality can potentially affect health outcomes “on average”, but also the health implications of these contextual factors among vulnerable groups, such as parents with unemployed children. We argue and empirically demonstrate the role of these contextual factors in limiting mental health disparities among older adults in India.

## 2 Theoretical framework

Children's unemployment can negatively impact parents' mental health through several mechanisms. Unemployment can disrupt family dynamics by increasing tensions within the family, leading to conflicts and strained relationships, which may further contribute to parental stress (Pillemer and Luscher, 2003). Another important channel might be financial strain, particularly in contexts where parents provide ongoing economic support to their unemployed adult children, which may increase their stress and anxiety (Umberson et al., 2010). This financial burden is often compounded by emotional distress, as parents with unemployed children may experience feelings of failure or guilt, worry about their children's future, or perceive their children's unemployment as a reflection of their own shortcomings as caregivers (Fingerman et al., 2012).

We focus on two theoretically and empirically grounded moderators to explore the heterogeneity in these intergenerational mental health associations: social participation and income inequality. Social participation serves as a proxy for the social capital and community integration available to older adults, and has been consistently linked to better health and reduced psychological distress (Bourassa et al., 2017; Levasseur et al., 2010). As such, it may buffer the psychological consequences of adult children's unemployment by offering emotional and instrumental support. Income inequality, on the other hand, captures broader societal-level processes that shape how unemployment is perceived and experienced. In more unequal societies, status competition and stigma related to joblessness are stronger (Wilkinson & Pickett, 2009), potentially amplifying the shame, stress, and social exclusion experienced by both the unemployed and their family members (Hatzenbuehler et al., 2013). By analysing these moderators, we aim to uncover not only whether adult children's unemployment is associated with parental mental health, but also under what conditions this link is mitigated.

### 2.1 Heterogeneity by social participation

Social participation may mitigate some of the mechanisms related to the negative impact of adult children's unemployment on parental mental health described above. Generally, social participation is seen as a key determinant of healthy ageing (Levasseur et al., 2010), as it encompasses all kinds of activities related to developing and maintaining a variety of social relationships and forms of involvement in the local community, such as interactions with neighbours and friends, as well as engagement in voluntary work and participation in local leisure and social activities. While adult children's unemployment may lead to tensions between children and parents, having frequent interactions with other relatives and friends may compensate for deteriorating intragenerational relations. By strengthening the social ties between older adults and the communities in which they live, social participation can help older adults access resources and support through their connections to others. These connections can be particularly important on “rainy days”, when older adults find themselves in circumstances in which their need for emotional or instrumental support is particularly acute.

Social connections may help older adults maintain lifestyles and habits that protect their mental health by enacting positive informal social control (Young et al., 2004). Social connections can also serve as an important channel for the provision of support by either providing relief to older adults who are at risk of experiencing poorer mental health or solving the problems that generated this risk in the first place. For instance, when older adults are exposed to their children's unemployment, their social connections can help them access emotional support to alleviate feelings of sadness, failure, or guilt, and prevent them from blaming their own shortcomings as parents for their offspring's unemployment (Fingerman et al., 2012). If unemployment causes disruptions in family dynamics, older adults' social connections may partly compensate for their missing contacts or even mediate any family conflicts they have with their adult children. Parents' social connections may also provide them with information about job opportunities, which can increase their adult children's chances of re-employment. Thus, receiving instrumental support may reduce parents' worries about their children's future and help them to re-establish feelings of control over their own and their family's economic situation.

While social participation has been seen as a protective factor for health, some studies also point to its potential negative aspects, especially when it comes to vulnerable population subgroups that are at risk of stigmatisation. Parental perceptions of their own failure to provide children with a good start in life may lead to social anxiety and anticipation of stigma (Dieckhoff & Gash, 2015; Kunze & Suppa, 2017). Therefore, one could argue that some of the parents with unemployed children might benefit relatively less from meetings with neighbours or friends if such meetings trigger feelings of shame instead of offering comfort, especially in societies with high stigmatisation of unemployment or status competition. This argument calls for special care in the analysis and interpretation of the results, a point to which we return to in the next sections.

While previous empirical research has shown that social participation has positive effects on health among older adults, we are not aware of any studies that test the role of social participation in buffering the consequences of adverse life events across generations. A study by Ang (2018) examining the development of social participation and health throughout the life course among Americans found that formal social participation has a protective effect on men's mental health as they age, while its effects remain consistent for women across all age groups. A longitudinal study on social participation in the European context also demonstrates that it is an important factor in cognitive functioning and healthy ageing (Bourassa et al., 2017). Lu et al. (2022) and Sirven and Debrand (2008) found that social participation leads to healthy ageing in the Japanese and European contexts. In summary, while social participation has been shown to improve population health, its role in reducing health inequalities has been largely overlooked.

### 2.2 Heterogeneity by income inequality

High income inequality in the state of residence may create a context in which losing a job is more economically damaging and increases the financial strain within a family. This is especially likely to occur in countries with strong intergenerational ties, such as India, where family members are obliged to provide ongoing economic support to each other, and adult children, in particular, are expected to provide financial support to their parents (Nagargoje et al., 2025). Moreover, income inequalities are often driven by the design of social policies (Polacko, 2021). Thus, in societies or regions with lower income inequality, families hit by unemployment tend to benefit from the cushioning role of unemployment benefits as well as active labour market policies (Voβemer et al., 2018).

Next to the mechanisms related to limited resources, state-level income inequality may imply stronger associations between adult children's unemployment and parental mental health due to the mechanisms of status competition and social capital (Rakesh et al., 2025). In Indian states with higher income inequality, the unemployment of adult children can intensify parental insecurities about their children's future prospects, as previous research has shown that parents in more unequal societies may feel heightened concern about their children's ability to compete for resources and opportunities (Wilkinson & Pickett, 2018). Furthermore, since negative societal perceptions of unemployment tend to be stronger in contexts with more pronounced income inequalities (Paul & Moser, 2009), in states with large disparities between the rich and the poor, having an unemployed family member may lead to feelings of status anxiety, stress, and shame. Lower state-level income inequality is linked with less stigmatisation of unemployment, and hence, in states with smaller disparities between the rich and poor, the associations between children's unemployment and parental mental health may be weaker. The mechanisms of status competition may be particularly relevant for older adults with high social participation. We will return to this point in the description of our data and methods.

## 3 Indian context

This study is particularly relevant to the Indian context, as India has the second-largest absolute number of older individuals in the world, despite having a relatively young population. Currently, there are approximately 140 million people aged 60 or older in India, accounting for about 10% of the total Indian population. This number is projected to increase to 320 million by 2050 (IIPS & UNFPA, 2023). The rapid ageing of the Indian population is concerning policymakers due to the substantial healthcare needs projected for the large elderly population in the future, especially as current levels of pension and healthcare benefits provided to older adults by the government are insufficient. India does not have a formal universal healthcare system, with a well-off population opting for private health insurance schemes with high premiums. In India, only 18% of the population aged 60 and above are covered by health insurance.

India has been a family-based culture where younger family members are still expected to provide informal care for older adults’ parents and grandparents. Thus, older adults in India heavily depend on their adult children for financial and healthcare support. Due to the prevalence of strong familial ties, adult children are expected to provide intergenerational financial support to their parents (Nagargoje et al., 2025). However, with changing family norms in contemporary India, there is a shift towards nuclear families and increasing outmigration to urban areas for work; therefore, the levels of healthcare and support that older adults receive are significantly reduced.

Son preference is also deeply rooted in Indian culture across generations and remains strongly prevalent in districts of the northern and central Indian states (Singh et al., 2022). This phenomenon of strong son preference has demographic and health implications as it leads to a skewed sex ratio at birth, favouring male children, and also poorer health outcomes and excess mortality for female children (Jayachandran, 2023). The latest round of the Demographic and Health Survey, conducted in India from 2019 to 2021, showed that 15% of respondents prefer to have more sons than daughters (International Institute for Population Sciences & ICF, 2021). This phenomenon of son preference is rooted in the traditional belief that sons carry over the family name and look after their parents in old age, whereas daughters leave for their matrimonial homes once married and incur the costs of a dowry. So, we can expect that an adult son's unemployment is more detrimental to parental mental health than an adult daughter's unemployment. This adverse effect of an adult son's unemployment will be more pronounced if it's the eldest son who becomes unemployed, as they are typically favoured in the Indian context due to the special role they play in taking care of the parents at older ages (Jayachandran, 2023).

The Indian economy has been growing rapidly over the past few decades, with growth exceeding 8% in some years of the 2000s. However, despite the strong economic growth, there has not been sufficient expansion of employment opportunities, especially for young people. The labour force participation in India is comparatively low at 55.4 percent for the population aged 15-64 (International Labor Organization & Institute for Human Development, 2024).

Indian youths have increasingly high educational attainment, but there are not enough employment opportunities created for them, as evidenced by the decreasing Labour force and working population in recent years. The current skills gap among Indian youth is also concerning, as a particularly worrying trend is that young people aged 15-29 with secondary or higher education comprised 65.7% of unemployed youth in 2022, compared to 35.2% in 2000 (International Labor Organization & Institute for Human Development, 2024). The report, co-authored by the ILO and the Institute for Human Development (IHD), highlights the dual issue faced by the Indian economy: a lack of employable skills among today's youth, and the Indian economy is also struggling to create quality jobs for them.

Public unemployment benefits schemes are scarce in India, and only factory workers, insured under the Employees' State Insurance Corporation can avail themselves of these benefits, which provide 50% of their income for 12 months. The income inequality in Indian states is also high, which has further risen from 2004 to 05 to 2011-12 (Tyagi, 2023). This creates a dual concern for older adults as they worry about the effects of unemployment on their children's well-being in a precarious employment situation in India (having uncertain and unstable jobs with fewer social benefits and statutory protections from the welfare system); in addition, they may be concerned about whether their children will be able to provide for their financial and healthcare needs as they depends on it.

## 4 Data & methods

### 4.1 The Longitudinal Ageing Study in India

For this study, we use data from the Longitudinal Ageing Study in India's (LASI) first available wave (2017-18), which includes 73,396 adults aged 45 and above, conducted across all 28 states and 8 Union Territories in India. Therefore, our data will be cross-sectional in nature. LASI employed a multistage cluster sampling design, utilising a three-stage sampling design in rural areas and a four-stage sampling design in urban areas. LASI collects information directly from all older adults, including their health, socioeconomic status, and well-being, for those aged 45 and above residing in the sampled household. They report information on their children living away, while details on children living in the same household can be obtained from the household roster, which provides information on all household members.

Older adults aged 45 and above are asked if their children don't reside with them about whether their children are currently studying, employed, self-employed, unemployed, or have some other status. We coded adult children who were studying or in the “Others” category as missing. We generate the combined children's unemployment indicator for parents using the binary employment status variable of the first eight adult children (children aged 21 and above). The indicator is coded as zero if all the children are employed and is coded as one if any of the first eight adult children are unemployed. We identify 54,583 older adults with children who have no unemployed adult children according to this indicator, and 15,258 older adults with at least one unemployed adult child. There are 3555 older adults in the data with no children who are excluded from our descriptive analysis. Thus, our exposure variable is constructed for older adults with at least one child over 21 who are unemployed, and the control group consists of older adults with at least one child. Table A.1 illustrates the differences in socio-demographic characteristics between older adults without children in the survey and those with children. We can clearly observe that the group of older adults without children are at a higher risk of depression (33.2 %) than the group of older adults with children (24.7 %). This is in line with a recent study on India, which also found that childlessness is associated with a higher risk of depression among adults in mid and later adulthood (Vikram et al., 2025).

Employment information on children residing in the same household as older adults is obtained from the survey's household questionnaire. For children living with their elderly parents, we obtain employment information by matching their socio-demographic characteristics from household roster files. This is done based on their household member ID, where we have information on each household member as to whether they are engaged in business, salaried, or agricultural work. We then coded the children's employment status into a binary indicator, assigning a value of zero to employed or self-employed individuals and a value of one to unemployed individuals. We provided a distinction in Table A.3 between the proportion of unemployed adult children measured directly and indirectly, by examining birth order-specific unemployment. For example, out of 9153 unemployed first-born children, 4506 (almost 49%) were living in the same household as their parents. For second-born children out of a total of 8461 unemployed children, 3443 (almost 41%) were living with their parents.

Our primary exposure variable is “any unemployed adult child among the first eight children”, and to uncover heterogeneity of our exposure, we further explore the exposure of adult children's unemployment by:

1. Number of unemployed children;
2. Gender- and birth-order-specific exposure (firstborn/second-born son vs daughter);
3. Co-residence status of children with parents.

We must turn to gender and birth order-specific analyses to examine differences in results by children's gender, as our primary exposure variable, “any unemployed adult child among the first eight children,” cannot specify whether a son or a daughter is unemployed in cases where both sons and daughters are unemployed. Similarly, for differences in results by children's co-residence status with their parents, we have to use birth order-specific analyses to differentiate the co-residence status of the children.

### 4.2 Mental health

**Fig. 1:**
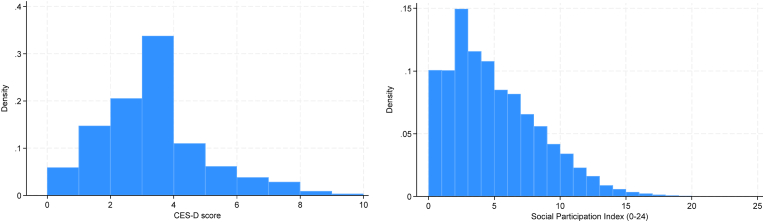
Distribution of the CES-D score and the Social Participation Index in LASI 2017-18.

We measure parental mental health using the CES-D score, with respondents reporting four or more symptoms out of 10 considered at the risk of being “depressed”. CES-D is a short self-report scale designed as a screening tool for depressive symptoms in the general population (Radloff, 1977). The original CES-D scale is a 20-item scale, but LASI uses a shortened 10-item CES-D scale, which has also been validated by several studies for measuring self-reported depression (Mohebbi et al., 2018; Zhang et al., 2012). We measure parental mental health using the CES-D score out of 10. The 10 items include: seven negative symptoms (trouble concentrating, feeling depressed, low energy, fear of something, feeling alone, bothered by things, and everything is an effort), and three positive symptoms (feeling happy, hopeful, and satisfied). For negative symptoms, the rarely or never (<1 day) and sometimes (1-2 days) categories are scored as zero, while the often (3-4 days) and most or all of the time (5-7 days) categories are scored as one. The scoring is reversed for the positive symptoms. Fig. 1 shows the distribution of the CES-D score in our sample. Around 25% of the elderly individuals in our sample have four or more symptoms out of 10, which are considered at “risk of depression” in our analysis.

### 4.3 Social participation

We construct the social participation of older adults variable based on the measure developed by Rana et al. (2022). The indicator is based on the responses to six questions on the frequency of (a) attending organisations, clubs, or social meetings/gatherings; (b) visiting relatives/friends; (c) attending cultural performances, shows, or the cinema; (d) attending religious functions/events such as bhajan, Satsang, or prayer; (e) attending political, community, or organisation group meetings; and (f) meeting with friends. The frequency of attending/visiting/meeting is coded as zero for never, one for at least once a year, two for at least once a month, three for at least once a week, and four for daily. The social participation score is the sum of all these codes, ranging from 0 to 24. Fig. 1 also shows the distribution of the Social Participation Index in our sample. Furthermore, we divide this measure into three tertiles: i.e., first (0-2), second (3-5), and third tertiles (6+) in our empirical analysis.

### 4.4 Income inequality

**Fig. 2:**
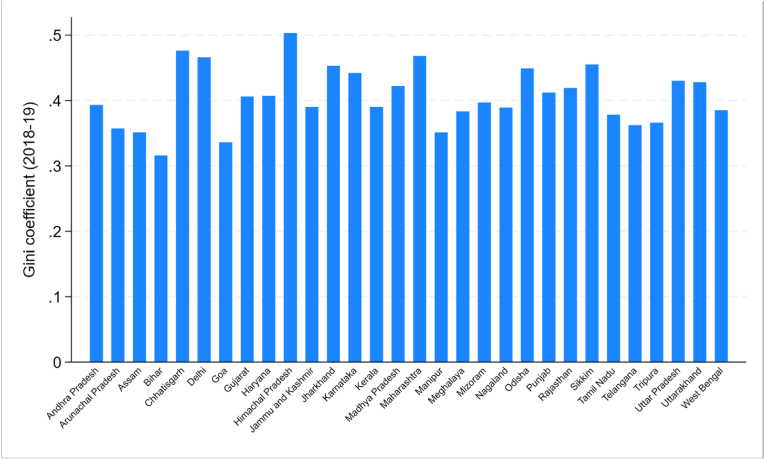
Distribution of the Gini coefficient across Indian states from PLFS 2018-19 data **Source:** Calculations by Chandrasekhar et al. (2021) from PLFS 2018-19 data.

We examine whether the relationship between children's unemployment and parents' mental health differs by income inequality at the state level, measured using the Gini coefficient at the state level calculated by Chandrasekhar et al. (2021) from PLFS 2018-19 data, as shown in Fig. 2. We divide the states into three equal tertiles based on their Gini scores, i.e., low-inequality states, medium-inequality states, and high-inequality states.

### 4.5 Statistical analyses

To account for confounders in our study, we use the weighting estimator approach in our observational data. Accounting for potential confounding is important because parents' depression risk could be selected based on characteristics such as low education, poor job prospects, and poor health, which, in turn, tend to predict their children's likelihood of being selected into unemployment. We employ the inverse probability weighting (IPW) approach to estimate the Average Treatment Effect on the Treated (ATET) of children's unemployment on the probability of depression in older adults, using a logistic regression model. We used ATET as the estimand in our study, as it leads to a clearer interpretation of older adults' mental health decline that actually corresponds to the older adults who experienced their children's unemployment. While using ATE, we will observe how the effect appears in the entire population, which may differ from the true effect due to the selection bias of older adults whose adult children become unemployed. Therefore, the estimand used throughout the study is the Average Treatment Effect on the Treated (ATET) for older adults with at least one unemployed adult child. To explore the heterogeneity of our exposure, we used different specifications of exposure, as explained in the Data and Methods section on page 11. However, our estimand remains the same, i.e., ATET. The IPW model used throughout the study only considers complete case analysis, i.e. only considers individuals with information on all covariates. Table A.4 presents the descriptive characteristics for our main IPW model, including those for which we obtained results and those observations that were dropped due to the presence of missing covariates. Our weighting model incorporates covariates that may confound the relationship between children's unemployment and parental mental health, i.e. individuals' characteristics, including age, sex, education, monthly per capita consumption expenditure (MPCE), multimorbidity, work status, self-reported health (SRH), marital status, number of children, and living arrangements. The IPW model only considers complete case analysis, i.e., it only considers individuals with information on all covariates. We used the treatment effects estimator, specifically the IPW estimator in Stata (teffects ipw), which by default provides robust standard errors when reporting the Average Treatment Effect on the Treated (ATET).

This IPW method first calculates the probability of being treated^2^ using a logistic regression model. Thus, we first model adult children's unemployment status using logistic regression. We then calculate the probability of being treated for older adults, i.e., having at least one unemployed adult child. Finally, we assign weights to the control group based on the inverse of the probability of being treated.

Adultchildrenemployment(i)=α(i)+βage(i)+β1sex+β2education(i)+β3MPCE(i)+β4multimorbidity(i)+β5workingstatus(i)+β6SRH(i)+β7Maritalstatus(i)+β8Livingarrangements(i)+β9No.ofchildren(i)+E(i)

Applying this generated weight helps to create the pseudo-treatment and control groups balanced on the characteristics of older adults that can predict their children becoming unemployed. IPW uses the propensity score to generate weights for the treated and control groups by weighting each individual according to their probability of receiving treatment. However, to generate ATET weights for IPW, we only reweight the control group in the following way: [Weight (IPW) = 1 for the treated group] and [Weight (IPW)= Propensity Score/(1-Propensity Score) for the control group]. Once the pseudo-population for the control groups has been created by reweighting the population using our weights generated by IPW, we run a weighted linear probability regression model on adult children's unemployment using the generated weights to compute our ATET.

$$y_{i}=\beta_{0}+\beta_{1}x_{i}+\epsilon_{i}$$

$y_{i}\;:\;Binary\;classification\;based\;on\;respondent\;i\;CES-D\;score\geq 4\;coded\;as\;1\;and<\;4\;coded\;as\;0.$

$x_{i}:Combined\;adult\;children^{\prime }s\;employment\;status\;of\;respondent\;i$.

**Fig. 3:**
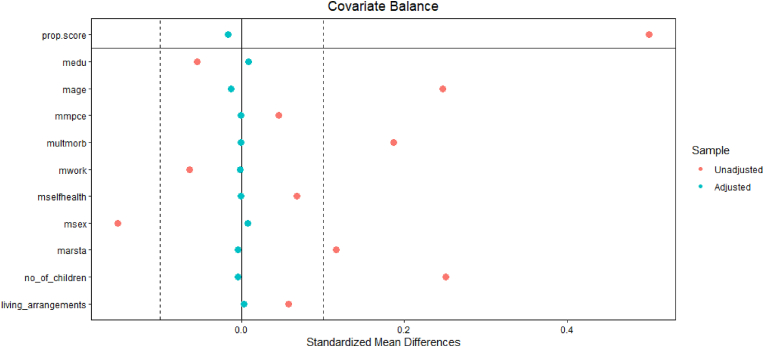
Absolute standardised mean differences for covariates, unadjusted, and adjusted after weighting using IPW.

We also check the suitability of IPW for balancing the covariates across groups after the procedure. Fig. 3 shows the absolute standardised mean differences for covariates before and after balancing based on IPW. We see that weighting through IPW balances the covariates across the treatment and control groups. In our heterogeneity analysis with social participation and income inequality at the state level, we used the state's Gini coefficient as described above. We run our analyses separately on subsamples of these categories of states, i.e., low, medium, and high inequality states. Similarly, we examine the heterogeneous effects of older adults' social participation on this relationship by conducting our analyses separately for three categories of social participation: low, medium, and high.

### 4.6 Multilevel modelling combined with IPW weights

The heterogeneity analysis of older adults' social participation level on the relationship between adult children's unemployment and older adults' depression risk could be influenced by their partner's mental health, and second, by the socio-economic landscape of their state, as higher social participation in poorer and high inequality states may exacerbate rather than ameliorate negative influences of adult children's unemployment on older adults' depression risk. To account for the clustering of results at the couple and state levels, we employ multilevel models that cluster at both the household and state levels, combined with IPW ATET weights, as in Bian et al. (2024). To achieve this, we first manually calculated the IPW ATET probability weights using a logistic regression model. We then run the weighted mixed-effects models, interacting adult children's unemployment with social participation levels and predicting average marginal effects for three categories of social participation, accounting for random slopes and random intercepts for state, as well as random intercepts for household ID. We also include state-level economic indicators, such as the Gini coefficient, unemployment rate, and monthly per capita household income, as calculated by Chandrasekhar et al. (2021) using PLFS data from 2018 to 19.

In addition to accounting for the clustering of results at the couple and state levels, the multilevel approach provides the opportunity to control for both social participation and state-level income inequality. As argued in our literature review, while social participation has been viewed as a protective factor for health, some studies suggest weaker or even negative effects in contexts of high inequality, where low social status is likely to be stigmatising (Dieckhoff & Gash, 2015; Kunze & Suppa, 2017). At the same time, higher income inequality may be more harmful for parents with unemployed children who have higher levels of social participation. While the IPW approach implemented at the first stage of our analyses requires comparing estimates within categories of one specific source of heterogeneity at a time, the multilevel approach implemented in the next step allows including both social participation and state-level income inequality simultaneously.

Finally, we perform robustness checks to examine the varying effects of adult children's characteristics, specifically gender, co-residency, and the number of unemployed children. We have created children's order-specific variables for heterogeneity analysis, as our main exposure variable, children's unemployment, defined as any of the first eight children being unemployed, couldn't capture the gender and socio-economic characteristics of unemployed children. Hence, we have to define children's order-specific unemployment, so we created these three variables: (i) children's gender and order-specific variables, i.e., first children's unemployment and second children's unemployment. i.e., first children's unemployment, where firstborn male (female) children's unemployment is compared to that of firstborn children who were employed. Similarly, we defined second children's unemployment based on the gender and employment status of the second-born children, to identify gender-specific effects; (ii) children's co-residence status for first and second children by gender; and (iii) the variable capturing the number of unemployed children for older adults is created as follows: 0 for no unemployed children, 1 for one unemployed adult child, 2 for at least two unemployed children simultaneously. We also conducted several robustness checks to test whether there is an association between the moderators, such as older adults' high social participation levels, or children's cohabitation statuses, with their risk of depression. We also test whether older adults residing with adult children are more likely to have higher social participation.

## 5 Results

### 5.1 Main findings

The descriptive findings in Table 1 reveal the differences in the socio-economic and demographic characteristics of the older adults in the two groups. The group with at least one unemployed adult child is negatively selected, as it has higher proportions of older adults, lower education levels, and greater multimorbidity compared to the group of older adults with no unemployed adult children. The older adults in this group are also more likely to be widowed and living alone compared to the older adults with no unemployed adult children. We can also see that 23.7% of the older adults in the control group who do not have an unemployed adult child are at risk of depression, compared to 28.2 % of the older adults with at least one unemployed child.

**Table 1:** Descriptive characteristics of older adults across different groups (i.e., those whose adult children are employed and those with at least one unemployed adult child).

	Employed	Unemployed	Total	Difference
	N (%)	N (%)	N (%)	(Chi-2 p value
**N**	54,583 (78.2%)	15,258 (21.8%)	69,841 (100.0%)	

**Risk of depression**				
No	41,079 (76.3%)	10,734 (71.8%)	51,813 (75.3%)	<0.001
Yes	12,768 (23.7%)	4224 (28.2%)	16,992 (24.7%)	

**Gender**				
Male	31,139 (57.0%)	9215 (60.4%)	40,354 (57.8%)	<0.001
Female	23,444 (43.0%)	6043 (39.6%)	29,487 (42.2%)	

**Education level**				
No education	25,111 (46.0%)	7105 (46.6%)	32,216 (46.1%)	<0.001
Primary education	13,036 (23.9%)	4165 (27.3%)	17,201 (24.6%)	
Secondary education	10,539 (19.3%)	2921 (19.1%)	13,460 (19.3%)	
Higher education	5895 (10.8%)	1067 (7.0%)	6962 (10.0%)	

**Age group**				
45-59	26,818 (55.3%)	6097 (41.2%)	32,915 (52.0%)	<0.001
60-69	13,322 (27.5%)	5008 (33.8%)	18,330 (29.0%)	
70-79	6116 (12.6%)	2687 (18.1%)	8803 (13.9%)	
80-84	1272 (2.6%)	584 (3.9%)	1856 (2.9%)	
85+	958 (2.0%)	432 (2.9%)	1390 (2.2%)	

**MPCE quintile**				
Poorest	10,863 (19.9%)	2927 (19.2%)	13,790 (19.7%)	<0.001
Poorer	11,122 (20.4%)	2969 (19.5%)	14,091 (20.2%)	
Middle	11,069 (20.3%)	2987 (19.6%)	14,056 (20.1%)	
Richer	10,966 (20.1%)	3214 (21.1%)	14,180 (20.3%)	
Richest	10,563 (19.4%)	3161 (20.7%)	13,724 (19.7%)	

**Multimorbidity**				
No multimorbidity	31,160 (57.1%)	7192 (47.2%)	38,352 (54.9%)	<0.001
1 multimorbidity	14,465 (26.5%)	4395 (28.8%)	18,860 (27.0%)	
2+ multimorbidity	8933 (16.4%)	3660 (24.0%)	12,593 (18.0%)	

**Working status**				
Never worked	15,258 (28.0%)	5681 (37.3%)	20,939 (30.0%)	<0.001
Currently working	26,759 (49.1%)	4886 (32.1%)	31,645 (45.3%)	
Currently not working	12,527 (23.0%)	4674 (30.7%)	17,201 (24.6%)	

**Self-reported health**				
Excellent	2372 (4.4%)	525 (3.5%)	2897 (4.2%)	<0.001
Very Good	11,114 (20.5%)	2743 (18.2%)	13,857 (20.0%)	
Good	21,359 (39.4%)	5877 (39.0%)	27,236 (39.4%)	
Fair	14,369 (26.5%)	4230 (28.1%)	18,599 (26.9%)	
Poor	4931 (9.1%)	1680 (11.2%)	6611 (9.6%)	

**Marital status**				
Married	43,512 (79.7%)	10,999 (72.1%)	54,511 (78.1%)	<0.001
Widowed	10,129 (18.6%)	3970 (26.0%)	14,099 (20.2%)	
Divorced or separated	941 (1.7%)	289 (1.9%)	1230 (1.8%)	

**Living arrangements**				
Living alone	1412 (2.6%)	484 (3.2%)	1896 (2.7%)	<0.001
Living with spouse	7071 (13.0%)	2135 (14.0%)	9206 (13.2%)	
Living with spouse & children	35,800 (65.6%)	8629 (56.6%)	44,429 (63.6%)	
Living with children	9060 (16.6%)	3641 (23.9%)	12,701 (18.2%)	
Living with children & others	1240 (2.3%)	369 (2.4%)	1609 (2.3%)	

**Number of children**				
1 child	5243 (9.6%)	758 (5.0%)	6001 (8.6%)	<0.001
2 children	14,275 (26.2%)	2881 (18.9%)	17,156 (24.6%)	
3 children	13,678 (25.1%)	3822 (25.0%)	17,500 (25.1%)	
4 children	9413 (17.2%)	3028 (19.8%)	12,441 (17.8%)	
5+ children	11,961 (21.9%)	4769 (31.3%)	16,730 (24.0%)	

**Fig. 4:**
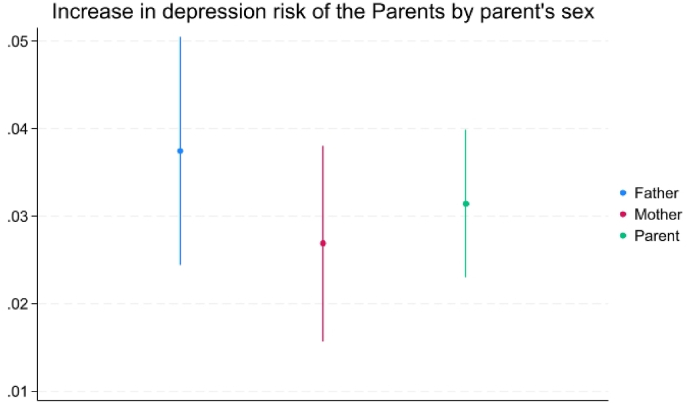
Average treatment effect on the treated (ATET) of children's unemployment on the probability of depression for older adults with 95% CIs by the parents' sex. **Note:** Average treatment effect on the treated (ATET) based on IWP.

The treatment effects ATET based on the IPW model in Fig. 4 reveal that adult children's unemployment is associated with an increase of 3.14 percentage points (ppts) in the probability of parental depression (12.48 % relative increase in the risk of depression). Interestingly, our results displayed in Fig. 4 also show no significant differences in the increased risk of depression between fathers (3.75 ppts, 16.16 % increased risk of depression in relative terms) and mothers (2.69 ppts, 10.13 % increased risk of depression in relative terms). We have included Table A.2 in the appendix, which shows these marginal effects with 95% Confidence Intervals and the number of observations for these analyses. The latter observation contrasts with findings from the UK (Albertini and Piccitto, 2023), indicating that mothers are more affected than fathers. This may be related to cultural differences in family dynamics and gender roles between India and Western countries.

**Fig. 5:**
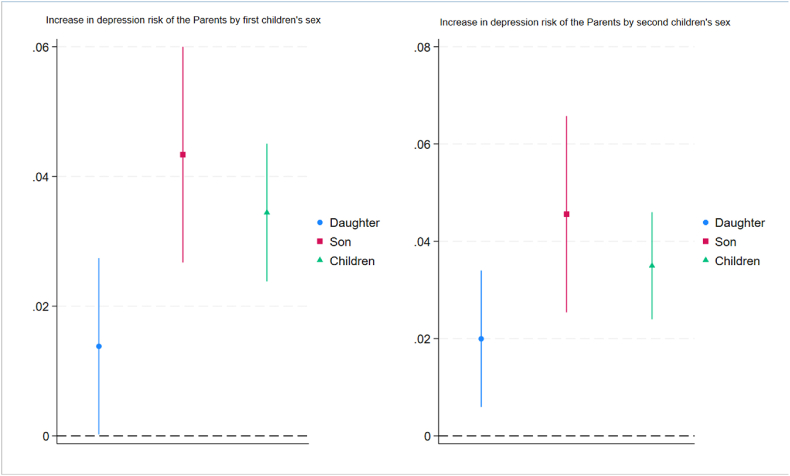
Average treatment effect on the treated (ATET) of children's unemployment on the probability of depression for older adults with 95% CIs by (i.) the first unemployed children's sex (ii.) the second unemployed children's sex **Note:** Average treatment effect on the treated (ATET) based on IPW.

Fig. 5 also reveals the increased depression risk based on the gender and order of the children, i.e., first or second children who are unemployed. We find significant differences in the increased risk of depression for parents with first unemployed children, individually for an unemployed adult son (daughter) compared to an employed child, as the parents' depression risk associated with their firstborn son's unemployment is 4.58 ppts (18.7 % increased risk of depression in relative terms). In comparison, the depression risk for parents associated with their daughter's unemployment is 1.55 ppts (6.07 % increased risk of depression in relative terms). For second children's unemployment and parents' increased risk of depression, sons' unemployment is associated with a stronger depression risk than daughters' unemployment. However, we do not find significant differences in the ATET estimates for sons and daughters, as the confidence intervals of the estimated ATET for the second-born son's unemployment and the second-born daughter's unemployment overlap; therefore, we cannot infer that their difference is statistically significant.

### 5.2 Heterogenous effects

**Fig. 6:**
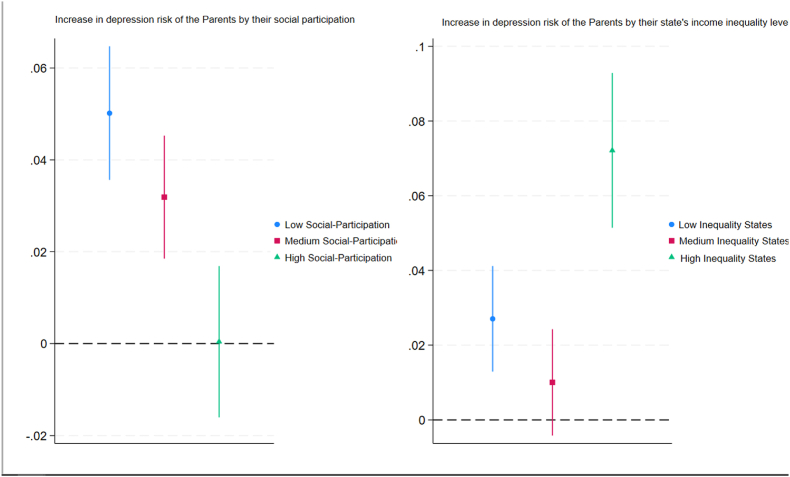
ATET of children's unemployment on the probability of major depression for older adults with 95% CIs by (i.) their social participation and (ii.) their state's income inequality level **Note:** Average treatment effect on the treated (ATET) based on IPW.

Our analysis in Fig. 6 indicates that for older individuals with high social participation levels, their adult children's unemployment has no significant association with their risk of depression (0.004 ppts increase), highlighting the importance of social connections and community engagement as buffers against stress and adverse life events. However, it is also evident that the confidence interval for the estimate of high social participation is the largest among other categories, suggesting that there might be contrasting effects of higher social participation on the depression risk of older adults. By contrast, older adults with low social participation levels with their adult children's unemployment are associated with an increased risk of depression by 4.93 ppts (17.87% increase in relative terms). This aligns well with findings from the broader literature on social capital and health (Kawachi et al., 2004). Note that the confidence intervals for the estimates by social participation are larger at higher levels of social participation, indicating that some older adults benefit more from social participation than others. However, the differences in older adults' depression risk associated with adult children's unemployment, across different social participation levels, disappeared when we accounted for clustering at the household and state levels and used the state's economic indicators in multilevel models in Figure A.12.

In addition, Fig. 6 shows that for older adults living in low and medium-inequality states, the increase in their risk of depression associated with their children's unemployment is weaker (2.62 ppts and 1 ppts increase, respectively) than that for older adults living in high-inequality states, who experience an increase of 7.21 percentage points (30% increase in relative terms). This suggests that living in a state with relative equality can act as a protective factor for individuals' mental health following their children's unemployment.

### 5.3 Robustness checks

**Fig. A.1:**
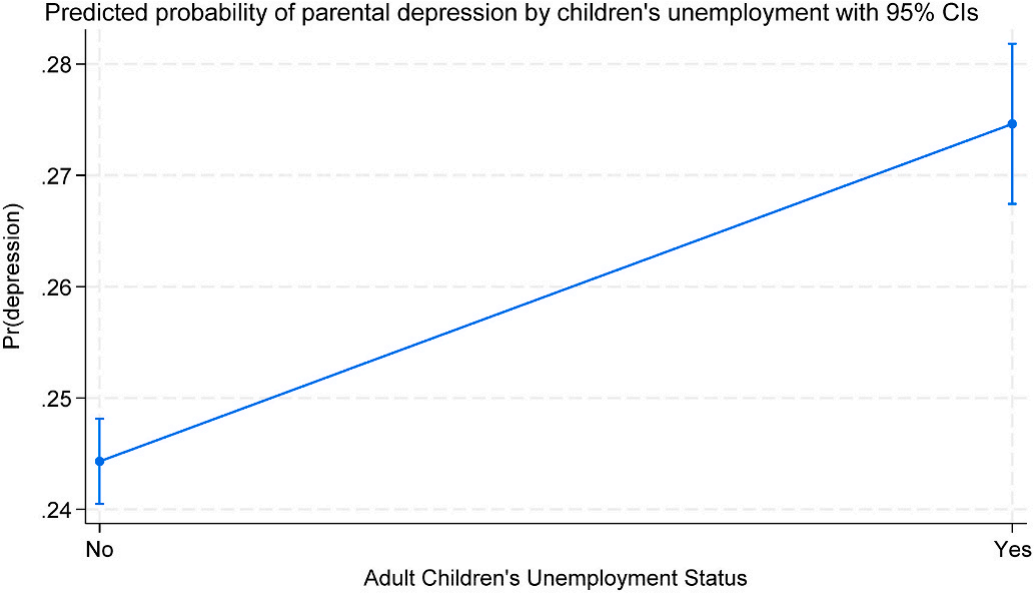
Predicted probability of depression (four or more symptoms) by children's employment status with 95% CIs.

**Fig. A.2:**
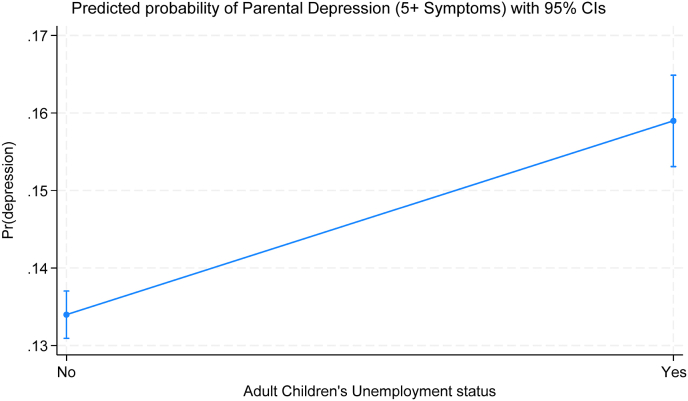
Predicted probability of depression (five or more symptoms) by children's employment status with 95% CIs.

**Fig. A.3:**
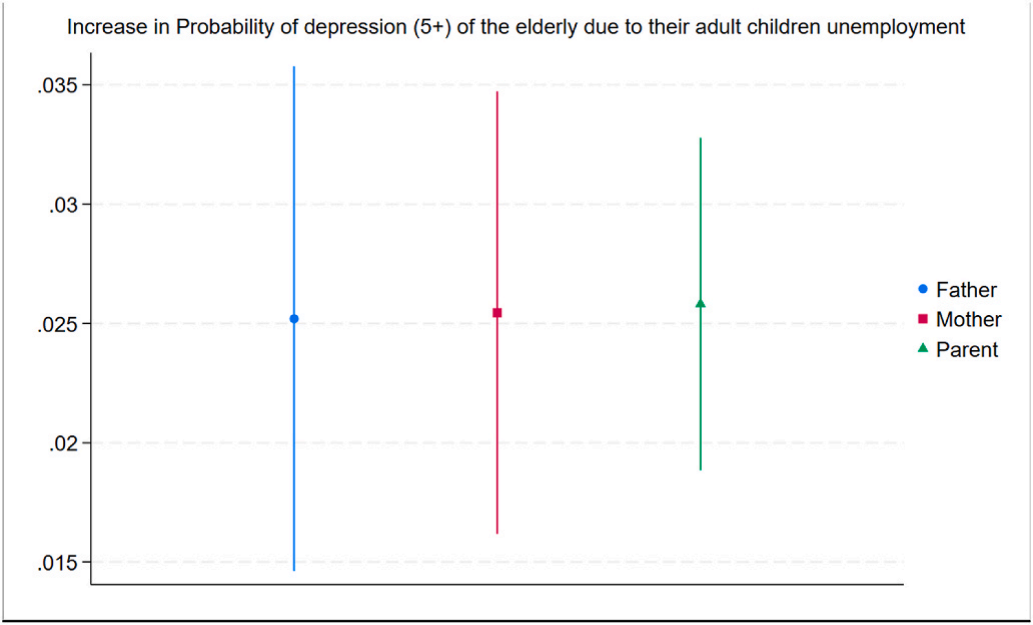
Average treatment effect on the treated (ATET) of children's unemployment on the probability of depression (five or more symptoms) for older adults with 95% CIs. **Note:** Average treatment effect on the treated (ATET) based on IPW.

**Fig. A.4:**
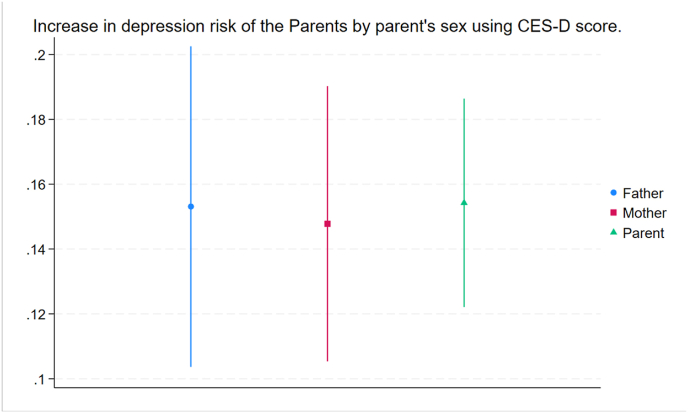
Average treatment effect on the treated (ATET) of children's unemployment on the probability of depression (continuous CES-D score) for older adults with 95% CIs. **Note:** Average treatment effect on the treated (ATET) based on IPW.

We ran several additional checks to ensure the robustness of our findings. Figure A.1 shows the predicted probability of depression risk with four or more symptoms by children's employment status with 95% CIs. Our usual measure of the risk of depression (four or more symptoms) shows a baseline probability of depression (0.244), which increases by around 0.031 to 0.275 for older adults with unemployed adult children (relative increase in the risk of depression of 12.3%). Our robustness measure in Fig. A.2, Fig. A.3 shows the predicted probability of depression risk (five or more symptoms). The risk of depression measure (five or more symptoms) shows a baseline probability of depression (0.134), which increases by around 0.025 to 0.159 for older adults with unemployed adult children (relative increase in the risk of depression of 18.5%). Thus, in both measures, there is an increase in the risk of depression within a similar range using the CES-D scale. We also run a similar analysis for older adults' depression risk with unemployed adult children using the continuous CES-D scale in Figure A.4, and find an increase of 0.148 in the CES-D score (relative increase in CES-D score by 5 %) in association with adult children's unemployment.

**Fig. A.5:**
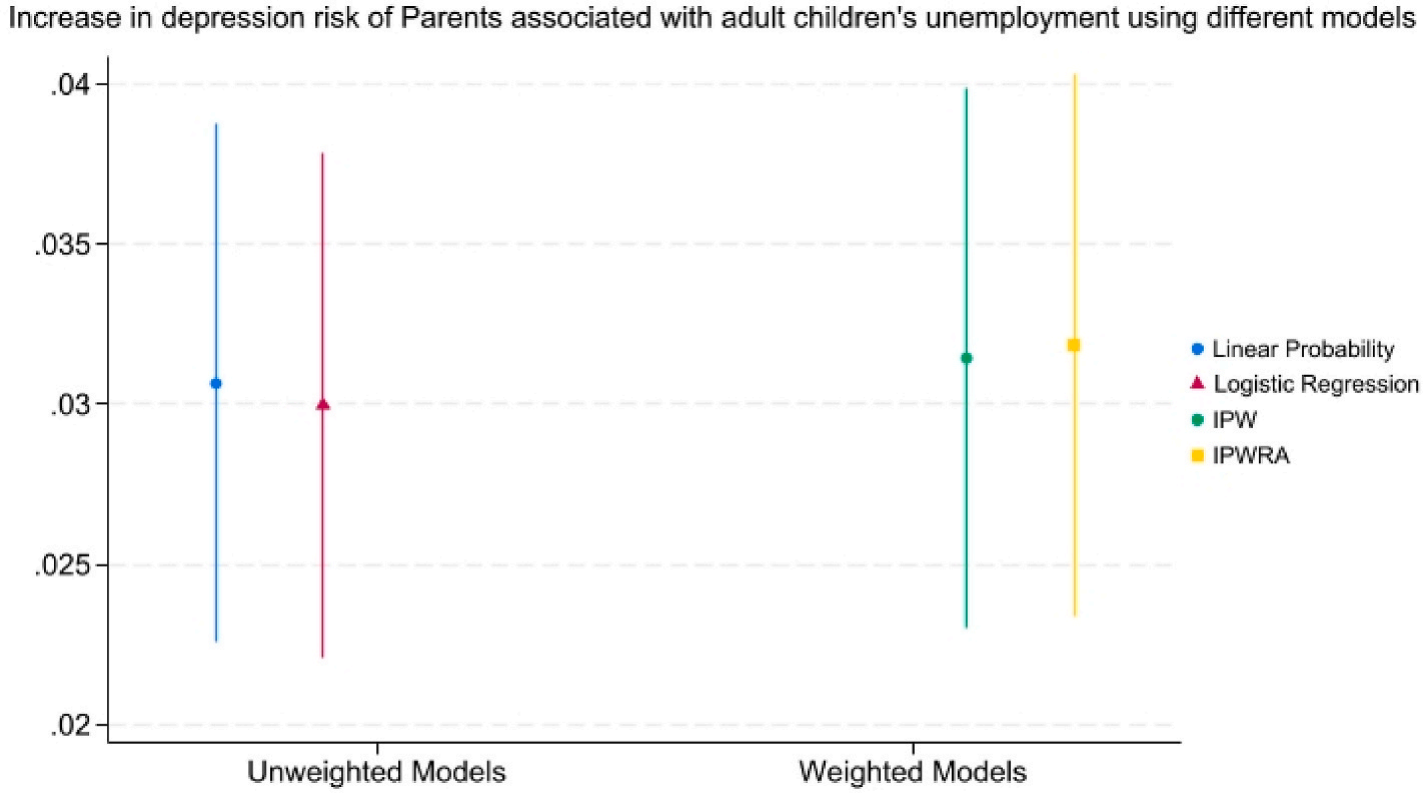
Average treatment effect on the treated (ATET) of children's unemployment on the probability of depression for older adults using IPW and IPW with regression adjustment 95% CIs.

**Fig. A.6:**
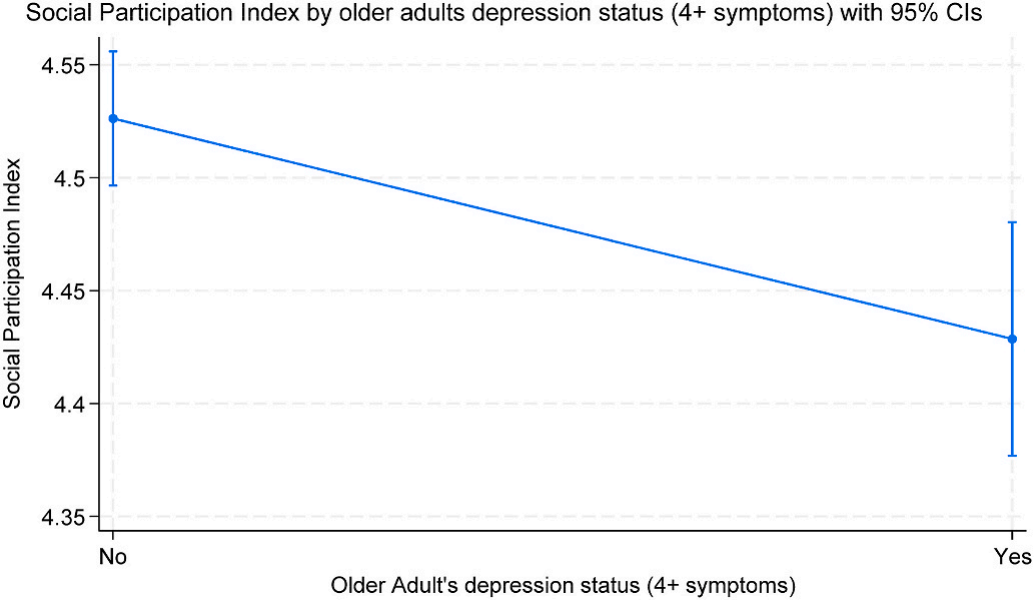
Association between the risk of depression and the Social Participation Index using OLS regression with 95% CIs.

**Fig. A.7:**
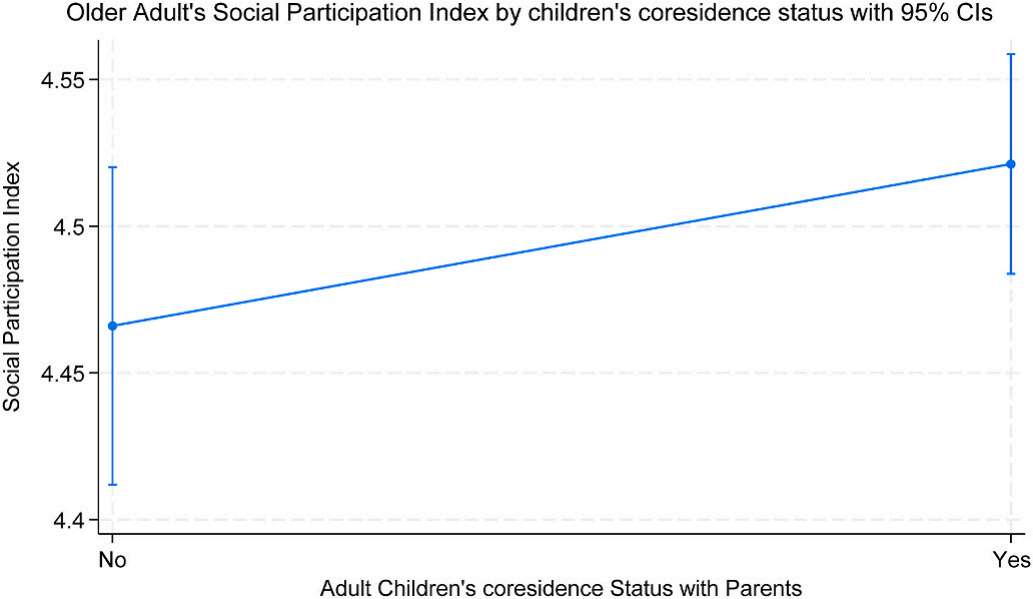
Association between adult children's co-residence with their parents and the Social Participation Index using OLS regression with 95% CIs.

**Fig. A.8:**
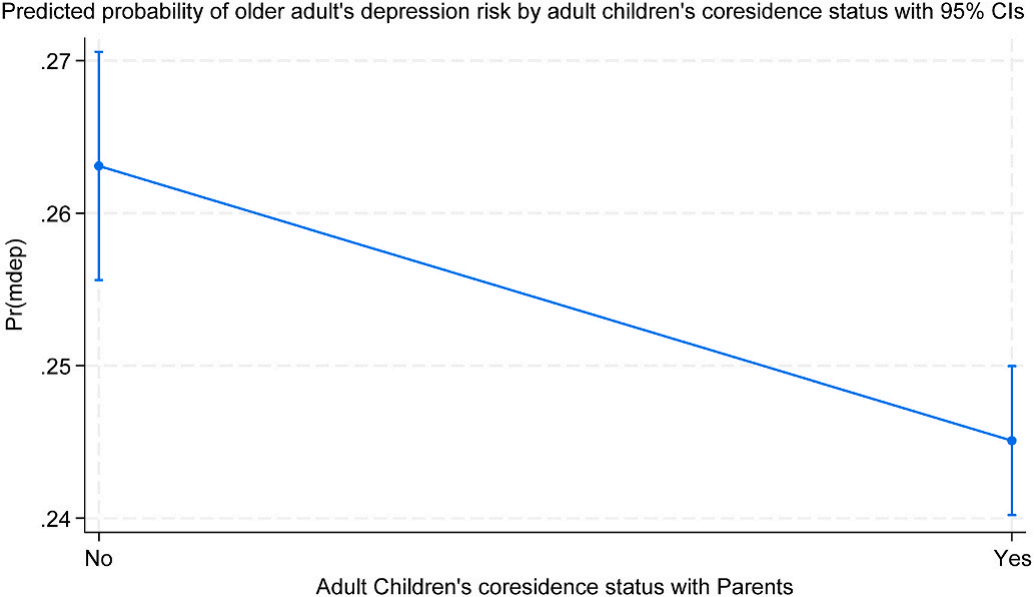
Association between adult children's co-residence with their parents and the risk of depression using OLS regression with 95% CIs.

The main results of our study use ATET from IPW throughout. However, we also estimate margins from unweighted models, such as linear probability and Logistic Regression models, with a similar set of covariates as the weighted models, including IPW and IPW with regression adjustment, as shown in Figure A.5, to test the robustness of our findings against potential confounding. This analysis, based on IPW with a regression adjustment function, fits an outcome model that includes covariates in addition to IPW weights generated using logistic regression. We also conducted robustness checks to test whether older adults with high social participation levels already have a lower risk of depression. Figure A.6 shows the association between the Social Participation Index and the risk of depression in older adults using OLS regression. There is a slight decrease from 4.52 for older adults who are not at risk of depression to 4.43 for older adults who are at risk of depression. Figure A.7 shows a non-significant increase in the Social Participation Index by children's co-residence status (if any of the first eight adult children co-reside with their parents). This confirms that older adults' social participation levels are independent of whether their adult children co-reside with them. Hence, the associations between older adults' depression risk and their children's unemployment by social participation levels are not confounded by their adult children's co-residence with them. However, Figure A.8 shows that the co-residence of adult children can potentially act as social capital for older adults by reducing their risk of depression from 0.263 to 0.245.

## 6 Discussion

Our study looked at whether adult children's unemployment is associated with parental mental health in India. Given that India has large inequalities in social capital and income, we examined how this relationship varies according to income inequality and social participation. Our findings show a 3.11 percentage points (ppts) increase in absolute terms (and a 12.30% relative increase) in the probability of parental depression risk associated with adult children's unemployment. This supports the “linked lives” principle (Elder Jr, 1998) by demonstrating that the poor socio-economic outcomes of adult children can be adversely linked to their parents' well-being, even in later life stages. Our findings on the overall negative association between children's unemployment and parental mental health are consistent with studies from the UK (Albertini and Piccitto, 2023), whereas a study from Europe suggested that this phenomenon does not follow a universal pattern, with the relationship varying depending on the welfare and the country's familial context (Bister et al., 2025). Interestingly, we observed no significant differences in the increased risk of depression associated with their children's unemployment between fathers (3.75 ppts, 16.16% increase in relative terms) and mothers (2.69 ppts, 10.13% increase in relative terms). Our results show a lack of gender differences for parental mental health in contrast with findings from the UK. However, we found significant differences in the magnitude of increased depression risks among parental mental health associated with their firstborn son's unemployment compared to their firstborn daughter's unemployment. This highlights the importance of considering the cultural context when examining intergenerational effects, as there is a strong son preference in India, especially for the eldest son, who is typically favoured in the Indian context due to the special role played by them in taking care of the parents at older ages (Jayachandran, 2023). Our finding that the unemployment of the eldest-born son is more detrimental than that of the eldest-born daughter aligns with the Indian context.

Our heterogeneity analysis revealed that among older adults with high social participation, there is no significant increase in the risk of depression associated with their children's unemployment, while there is a moderate increase in the association of depression risk among older adults with medium social participation and a much larger increase among older adults with low social participation (5.02 ppts, 18.23% increase in relative terms). However, the differences in older adults' depression risk associated with adult children's unemployment, across different social participation levels, disappeared when we accounted for clustering at the household and state levels and included the state's economic indicators in multilevel models. This could potentially mean that while there is heterogeneity by social participation for this relationship, this heterogeneity is driven by the household and state's contexts.

We also found that for adults in low and medium-income inequality states, the negative associations of their children's unemployment are much weaker than those for older adults in high-income inequality states, where the adult children's unemployment is associated with an increased risk of depression by 7.21 percentage points (30% increase in relative terms). Overall, our study indicates that having substantial social capital and living in a state with low-income inequality is associated with lower increases in risk of depression and can help to safeguard the mental health of older adults following their children's unemployment.

**Fig. A.11:**
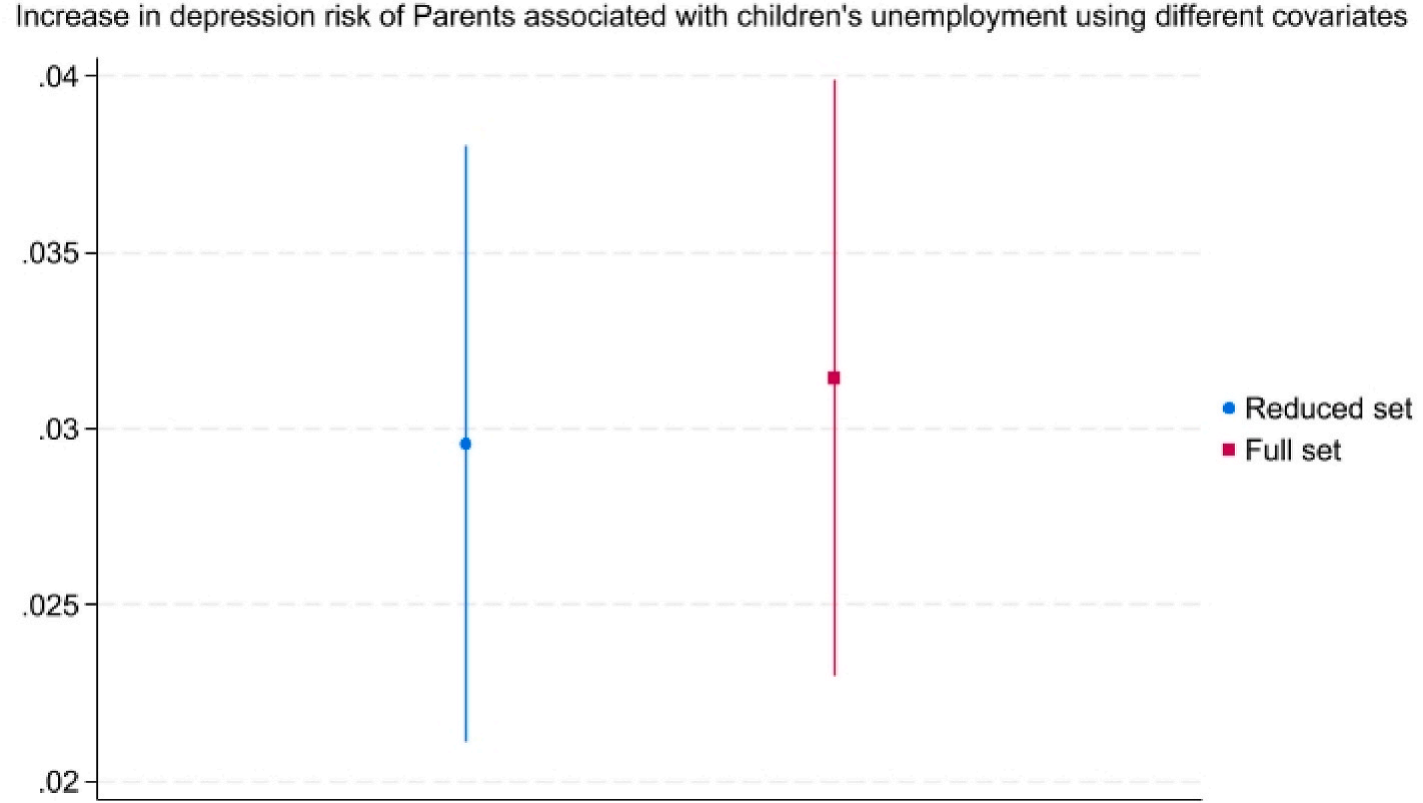
Average treatment effect on the treated (ATET) of children's unemployment on the probability of depression for older adults using IPW with different set of covariates and 95% CIs.

**Fig. A.12:**
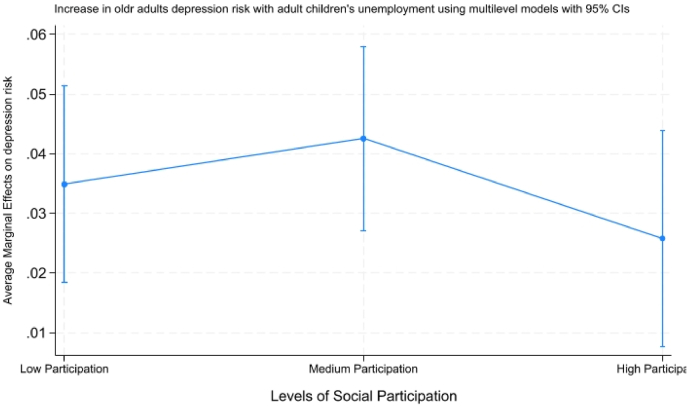
Average marginal effect of children's unemployment on depression risk for older adults across social participation levels using IPW and multilevel modelling with 95% CIs. **Note:** Average treatment effect on the treated (ATET) based on IPW and multilevel modelling.

It is important to note the limitations of our study. While we establish a strong association, causal inferences are limited due to the cross-sectional nature of the data. Longitudinal research designs using data from the next waves of LASI could provide stronger evidence of causal relationships (Kawachi et al., 1997). Our binary classification of employment status may not capture the nuances of underemployment or precarious employment, as we cannot account for the length of the unemployment spell, which could have heterogeneous effects on parental mental health, as shown by Albertini and Piccitto (2023) for the UK. Additionally, we lack information on the adult children's reasons for being unemployed, which could clarify whether their unemployment is involuntary or voluntary due to economic inactivity. Also, there might be children who left their jobs voluntarily to help their parents struggling with their health, so the temporal order of children's unemployment and parental depression cannot be established based on the data, especially for those residing in the same household as their parents, so we cannot claim a causal effect. However, we control for older adults' self-reported health and multimorbidity status to rule out the possibility of self-selection into unemployment among adult children based on their parents' health. However, accounting for older adults' health and working status in IPW might introduce post-treatment bias and result in conservative estimates. Therefore, we conducted a robustness check using two IPW models, estimating ATET for both models using IPW, with the first model using a smaller set of covariates and the second model with a standard set of covariates used throughout the study (including parental health and work status) in Fig A.11. We find almost identical ATETs for both models, meaning that including post-treatment variables throughout the study does not significantly bias our estimates and results.

Also, given the context of the labour market in India, it would be worthwhile to differentiate between employed adult children with diverging types of working conditions. Future studies could explore more detailed occupational classifications of children to gain insight into whether unemployment may be considered more favourable than working under stressful or harmful situations. Additionally, our measure of social participation did not distinguish between formal and informal types of social participation, which future studies should explore to determine which type of social participation is more beneficial for the protective effect.

Our unemployment measure can distinguish between unemployment, defined as not having a job and actively searching for one, and other forms of worklessness that are more voluntary in nature for children not living with parents we have their employment status from the individual questionnaire, but for children living together with their parents we estimate indirectly through household questionnaire where we add employment information for children living in the same household as their parents by checking whether they are engaged in business, salaried, or agricultural jobs, so we do not know for sure if they are not working voluntarily or involuntarily, so this is a limitation of our exposure variable children's unemployment. Therefore, there may be unobserved confounding in the relationship between adult children's unemployment and parents' mental health for children residing with their parents, as we cannot fully distinguish between voluntary and involuntary unemployment due to the indirect estimation of children's unemployment and the cross-sectional nature of the data. Therefore, there is a risk of misclassification bias, as children residing with parents may be unwilling to participate in the labour force by choice, and we may misclassify them as unemployed. We also cannot control for children's own health, as this information is not available in the data, which might also confound this association, as they may be unemployed due to their own health; at the same time, their parents might be at a higher risk of depression due to the children's poor health.

The cross-sectional nature of the data also limits us from ruling out reverse causality between adult children's unemployment and parental mental health, as children may voluntarily leave employment to care for less healthy or depressed parents; also, parental mental health may shape children's labour market outcomes through social and economic channels. There might also be unobserved confounding in our estimates for the relationship between adult children's unemployment and parental mental health, as children's unemployment is likely correlated with unobserved parental characteristics (e.g. long-run mental health, parenting style, early-life socioeconomic conditions) and also with their own unobserved characteristics (ability, health, motivation), which are missing in the data. Therefore, despite getting robust results across multiple specifications of the weighting model, such as IPW and IPW with regression adjustment, we cannot claim that our results are free from unobserved confounding. We can only claim that our results are independent of a particular functional form or different outcome variable definitions based on the CES-D scale.

The observed heterogeneity in associations between adult children's unemployment and parental mental health due to social participation may also be attributed to the selection of older adults into higher social participation levels, resulting from differences in their unobserved characteristics or contextual factors, rather than a direct protective mechanism for their mental health in the face of their adult children's unemployment. Also, the heterogeneity in the association by income inequality among states may not only reflect the heterogeneity resulting from income inequality, but the categorization of states into low, medium and high inequality based on the Gini coefficient of income inequality states can also reflect differences between the states in other dimensions than income inequality, in terms of state's capacity in delivering basic public services such as education, health, welfare and justice. Therefore, we cannot infer from our findings that reductions in state-level inequality would necessarily reduce the impact of children's unemployment on parental mental health, as it might be related to other differences among states, as explained above.

We also cannot provide evidence on the mechanisms leading to the increased parental risk of depression due to the cross-sectional nature of the data. Additionally, the financial and relational strains pathways for heterogeneity analysis by social participation and status competition pathways for heterogeneity analysis by income inequality could not be confirmed in our analysis, as these are hypothesised pathways consistent with prior literature, rather than tested mechanisms in this study. Future research could also explore geographic differences in more detail to provide a more fine-grained analysis of the large inequalities in India.

Despite its limitations, our study makes several contributions to the literature. To our knowledge, we are the first to provide evidence that adult children's unemployment is associated with an increased risk of parental depression in the Indian context. This finding aligns with our expectations, given that parents tend to be emotionally and financially interdependent with their adult children at older ages. Similar studies conducted in the UK and European contexts examined heterogeneities based on children's unemployment duration and the employment context, such as the unemployment rate or economic conditions. At the same time, we provided evidence that non-employment factors, such as high social participation or residing in a state with low-income inequality, could also shield the mental health of older adults following their children's unemployment. This protection may be due to their ability to find support in the community or through state government policies. Moreover, our findings are robust to the different specifications of the CES-D score for the risk of depression.

The lack of employable skills among today's youth in India, combined with the economy's inability to create quality jobs, raises concerns for older adults. In addition to worrying about the effects of unemployment on their children's well-being, parents may be concerned about whether their unemployed children will be able to provide for their own financial and healthcare needs. However, the implications of our results are not only limited to the Indian context but also to several LMICs and Upper Middle-Income Countries, with a high global youth unemployment rate of 12.6 percent in 2024, and the highest youth unemployment rate in upper-middle-income countries such as Brazil, Mexico, and China is 15.6 per cent (International Labour Office, 2025). Additionally, some of these countries will face population ageing issues in the future, as Brazil already has 10.9% of its population over 60, and China has 21.1% of its population over 60. Therefore, in the absence of robust social security for these ageing societies, they often depend on their adult children for support in old age.

Our findings regarding the protective effect of social participation suggest that interventions to enhance social connections among individuals could be beneficial for their mental health, particularly in the face of family stressors. This can be done by organising social activities for older adults, which could help increase social participation and build protective social capital. However, the policy implication of higher social participation associated with weaker associations for this relationship is valid only if the heterogeneous effects are established using a panel data setting to rule out selection into social participation and reverse causality arising from the incorrect temporal order of parents' mental health and social participation. The social security for older adults should also be strengthened, as the current reimbursements of National Pension Schemes are too low to sustain a dignified life without relying on their children. Additionally, our findings on the heterogeneous association for this relationship by state level income inequality suggest that effective state policies can provide economic security for older adults, including protection against adverse impacts from their children's life events. Supporting youth in employment is a crucial step in this process. Governments may consider expanding youth skills programs and employment guarantee schemes. However, these policy recommendations are only valid if the observed associations between adult children's unemployment and parental depression risk reflect causal processes, as only then policies that improve youth labour market prospects might also have beneficial implications for older adults' mental health. While it is possible to assess causal relationships using observational data with certain study designs, this isn't possible with cross-sectional data (Vanderweele, 2021). Therefore, due to the cross-sectional design of the study and potential confounding, this paper should not be interpreted as providing causal evidence that specific policies will improve parents' mental health. Therefore, more research should be conducted on this topic using Longitudinal research designs when data from the next waves of LASI are available to provide stronger evidence of causal relationships and more robust policy implications.

## CRediT authorship contribution statement

**Rishabh Tyagi:** Writing – review & editing, Writing – original draft, Visualization, Methodology, Formal analysis, Data curation, Conceptualization. **Anna Baranowska-Rataj:** Writing – review & editing, Writing – original draft, Methodology, Conceptualization. **Alexi Gugushvili:** Writing – review & editing, Writing – original draft, Methodology, Conceptualization.

## Preprints

This paper is out in the MPIDR Working Paper series.

Tyagi, R., Baranowska-Rataj, A., & Gugushvili, A. (2025). Adult children's unemployment and parental mental health in India: social and economic moderators (No. WP-2025-005). Max Planck Institute for Demographic Research, Rostock, Germany.

## Ethics approval statement

This study only uses secondary data, which is publicly available on request through the data request form; no ethical approval was required.

## Research data

Data is publicly available for academic research purposes on request through the data request form. The data can be requested from https://www.iipsindia.ac.in/content/LASI-data.

## Funding acknowledgements

Rishabh Tyagi was supported for his doctoral studies by the Max Planck Institute for Demographic Research, Rostock, Germany and the Research Council of Norway – project number 296297 (DIMJOB) and its Centres of Excellence funding scheme, project No. 262700. He also gratefully acknowledges the training and resources provided by the International Max Planck Research School for Population, Health and Data Science (IMPRS-PHDS).

Anna Baranowska-Rataj was supported by funding from the European Research Council (ERC) under the European Union's Horizon 2020 research and innovation programme under grant agreement No 802631.

## Declaration of competing interests

The authors declare that they have no known competing financial interests or personal relationships that could have appeared to influence the work reported in this paper.

## Figures and Tables

**Fig. A.9:**
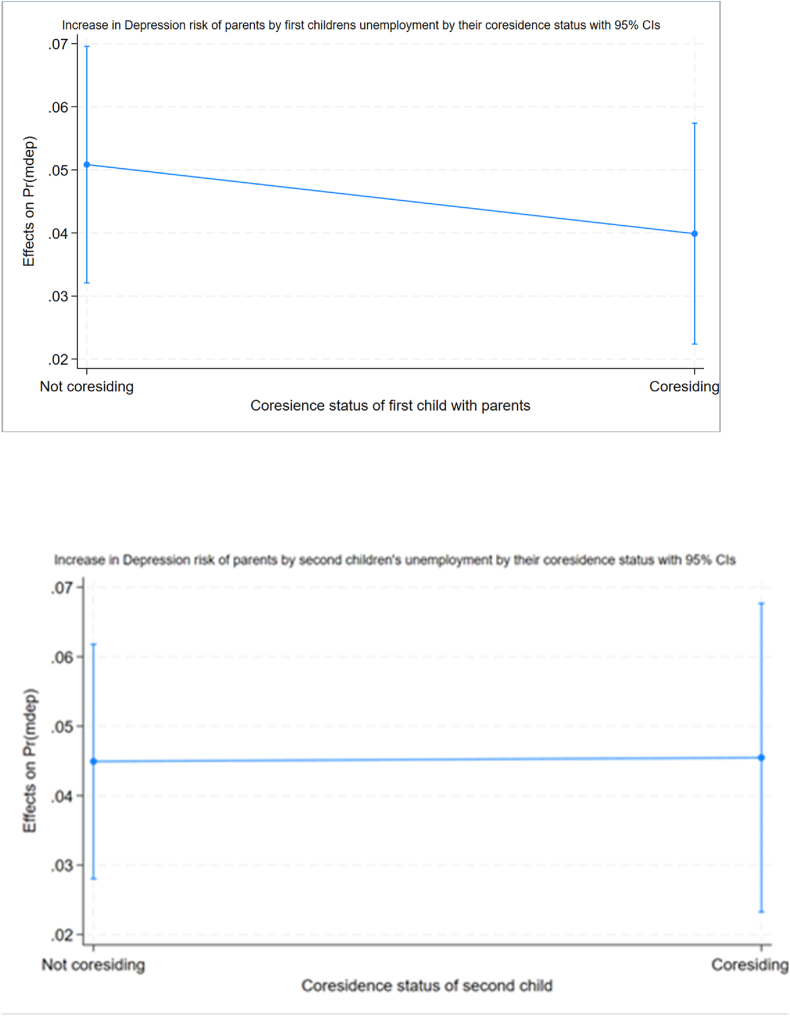
Association between adult children's unemployment and parents' depression risk by co-residence with their parents for (a.) first child and (b.) second children using Logit regression with 95% CIs.

**Fig. A.10:**
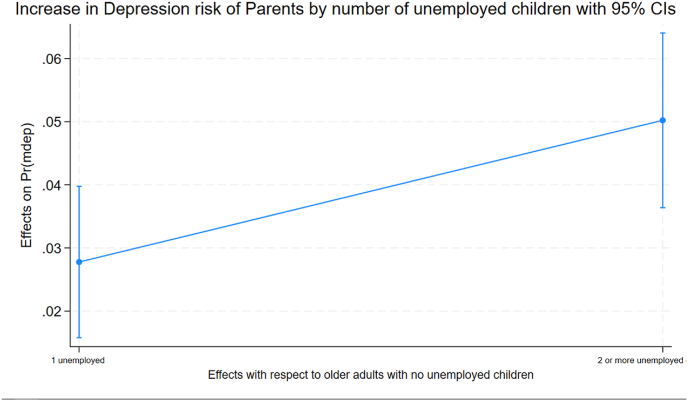
Association between adult children's unemployment and parents' depression risk by number of unemployed children using Logit regression with 95% CIs.

**Table A.1:** Descriptive characteristics of older adults across different groups (i.e., those who have children and those with no children)

	Employed	Unemployed	Total	Difference
	N (%)	N (%)	N (%)	(Chi-2 p value)
**N**	69,841 (95.2%)	3555 (4.8%)	73,396 (100.0%)	

**Risk of depression**				
No	51,813 (75.3%)	1800 (66.8%)	53,613 (75.0%)	<0.001
Yes	16,992 (24.7%)	896 (33.2%)	17,888 (25.0%)	

**Gender**				
Female	40,354 (57.8%)	1907 (53.6%)	42,261 (57.6%)	<0.001
Male	29,487 (42.2%)	1648 (46.4%)	31,135 (42.4%)	

**Education level**				
No education	32,216 (46.1%)	1542 (43.4%)	33,758 (46.0%)	<0.001
Primary education	17,201 (24.6%)	836 (23.5%)	18,037 (24.6%)	
Secondary education	13,460 (19.3%)	685 (19.3%)	14,145 (19.3%)	
Higher education	6962 (10.0%)	490 (13.8%)	7452 (10.2%)	

**Age group**				
45-59	32,915 (52.0%)	1789 (54.0%)	34,704 (52.1%)	0.011
60-69	18,330 (29.0%)	881 (26.6%)	19,211 (28.8%)	
70-79	8803 (13.9%)	447 (13.5%)	9250 (13.9%)	
80-84	1856 (2.9%)	117 (3.5%)	1973 (3.0%)	
85+	1390 (2.2%)	78 (2.4%)	1468 (2.2%)	

**MPCE quintile**				
Poorest	13,790 (19.7%)	632 (17.8%)	14,422 (19.6%)	<0.001
Poorer	14,091 (20.2%)	666 (18.7%)	14,757 (20.1%)	
Middle	14,056 (20.1%)	708 (19.9%)	14,764 (20.1%)	
Richer	14,180 (20.3%)	728 (20.5%)	14,908 (20.3%)	
Richest	13,724 (19.7%)	821 (23.1%)	14,545 (19.8%)	

**Multimorbidity**				
No multimorbidity	38,352 (54.9%)	1959 (58.0%)	40,311 (55.1%)	0.002
1 multimorbidity	18,860 (27.0%)	858 (25.4%)	19,718 (26.9%)	
2+ multimorbidity	12,593 (18.0%)	560 (16.6%)	13,153 (18.0%)	

**Working status**				
Never worked	20,939 (30.0%)	1009 (28.5%)	21,948 (29.9%)	0.164
Currently working	31,645 (45.3%)	1635 (46.2%)	33,280 (45.4%)	
Currently not working	17,201 (24.6%)	895 (25.3%)	18,096 (24.7%)	

**Self-reported health**				
Excellent	2897 (4.2%)	138 (4.2%)	3035 (4.2%)	0.373
Very Good	13,857 (20.0%)	647 (19.9%)	14,504 (20.0%)	
Good	27,236 (39.4%)	1290 (39.7%)	28,526 (39.4%)	
Fair	18,599 (26.9%)	835 (25.7%)	19,434 (26.8%)	
Poor	6611 (9.6%)	339 (10.4%)	6950 (9.6%)	

**Marital status**				
Married	54,511 (78.1%)	1772 (49.9%)	56,283 (76.7%)	<0.001
Widowed	14,099 (20.2%)	683 (19.2%)	14,782 (20.1%)	
Divorced or separated	1230 (1.8%)	1097 (30.9%)	2327 (3.2%)	

**Number of children**				
1 child	6001 (8.6%)	0 (0.0%)	6001 (8.2%)	<0.001
2 children	17,156 (24.6%)	0 (0.0%)	17,156 (23.4%)	
3 children	17,500 (25.1%)	0 (0.0%)	17,500 (23.8%)	
4 children	12,441 (17.8%)	0 (0.0%)	12,441 (17.0%)	
5+ children	16,730 (24.0%)	0 (0.0%)	16,730 (22.8%)	

**Living arrangements**				
Living alone	1896 (2.7%)	436 (12.3%)	2332 (3.2%)	<0.001
Living with spouse	9206 (13.2%)	1728 (48.6%)	10,934 (14.9%)	
Living with spouse & children	44,429 (63.6%)	0 (0.0%)	44,429 (60.5%)	
Living with children	12,701 (18.2%)	0 (0.0%)	12,701 (17.3%)	
Living with children & others	1609 (2.3%)	1391 (39.1%)	3000 (4.1%)	

**Table A.2:** Marginal effects and confidence intervals for the main and gender heterogeneity analyses.

	Full sample	Male sample	Female sample
**ATET**	0.031	0.037	0.027
	(0.004)	(0.007)	(0.006)

**95% CI**	[0.023 - 0.040]	[0.024 – 0.050]	[0.016 – 0.038]
	0.252	0.232	0.265
**Depression risk**	(0.002)	(0.004)	(0.003)
**N**	62190	28847	33343

**Table A.3:** Number and percentage of unemployed adult children measured directly and indirectly for first and second birth order.

	Unemployed child living outside HH (N%)	Unemployed child living in the same HH (N%)	Total (N%)
**First Child**	4647	4506	9153
	(51 %)	(49 %)	(100 %)

**Second Child**	5018	3443	8461
	(59%)	(41%)	(100%)

**Table A.4:** Descriptive characteristics of older adults across different groups (i.e., those who were included in the main model and those who were not included in the main model due to missing observations on one of the covariates).

	Dropped	Retained	Total	Difference
	N (%)	N (%)	N (%)	(Chi-2 p value
**N**	7643 (10.9%)	62,198 (89.1%)	69,841 (100.0%)	

**Risk of depression**				
No	5248 (79.4%)	46,565 (74.9%)	51,813 (75.3%)	<0.001
Yes	1359 (20.6%)	15,633 (25.1%)	16,992 (24.7%)	

**Gender**				
Male	7008 (91.7%)	33,346 (53.6%)	40,354 (57.8%)	<0.001
Female	635 (8.3%)	28,852 (46.4%)	29,487 (42.2%)	

**Education level**				
No education	2968 (38.8%)	29,248 (47.0%)	32,216 (46.1%)	<0.001
Primary education	1846 (24.2%)	15,355 (24.7%)	17,201 (24.6%)	
Secondary education	1932 (25.3%)	11,528 (18.5%)	13,460 (19.3%)	
Higher education	895 (11.7%)	6067 (9.8%)	6962 (10.0%)	

**Age group**				
45-59	331 (30.2%)	32,584 (52.4%)	32,915 (52.0%)	<0.001
60-69	263 (24.0%)	18,067 (29.0%)	18,330 (29.0%)	
70-79	249 (22.7%)	8554 (13.8%)	8803 (13.9%)	
80-84	111 (10.1%)	1745 (2.8%)	1856 (2.9%)	
85+	142 (13.0%)	1248 (2.0%)	1390 (2.2%)	

**MPCE quintile**				
Poorest	1457 (19.1%)	12,333 (19.8%)	13,790 (19.7%)	0.005
Poorer	1541 (20.2%)	12,550 (20.2%)	14,091 (20.2%)	
Middle	1540 (20.1%)	12,516 (20.1%)	14,056 (20.1%)	
Richer	1667 (21.8%)	12,513 (20.1%)	14,180 (20.3%)	
Richest	1438 (18.8%)	12,286 (19.8%)	13,724 (19.7%)	

**Multimorbidity**				
No multimorbidity	5217 (68.6%)	33,135 (53.3%)	38,352 (54.9%)	<0.001
1 multimorbidity	1639 (21.5%)	17,221 (27.7%)	18,860 (27.0%)	
2+ multimorbidity	751 (9.9%)	11,842 (19.0%)	12,593 (18.0%)	

**Working status**				
Never worked	3545 (46.7%)	17,394 (28.0%)	20,939 (30.0%)	<0.001
Currently working	2976 (39.2%)	28,669 (46.1%)	31,645 (45.3%)	
Currently not working	1066 (14.1%)	16,135 (25.9%)	17,201 (24.6%)	

**Self-reported health**				
Excellent	468 (6.7%)	2429 (3.9%)	2897 (4.2%)	<0.001
Very Good	1944 (27.8%)	11,913 (19.2%)	13,857 (20.0%)	
Good	2848 (40.7%)	24,388 (39.2%)	27,236 (39.4%)	
Fair	1398 (20.0%)	17,201 (27.7%)	18,599 (26.9%)	
Poor	344 (4.9%)	6267 (10.1%)	6611 (9.6%)	

**Marital status**				
Married	7134 (93.4%)	47,377 (76.2%)	54,511 (78.1%)	<0.001
Widowed	421 (5.5%)	13,678 (22.0%)	14,099 (20.2%)	
Divorced or separated	87 (1.1%)	1143 (1.8%)	1230 (1.8%)	

**Living arrangements**				
Living alone	41 (0.5%)	1855 (3.0%)	1896 (2.7%)	<0.001
Living with spouse	375 (4.9%)	8831 (14.2%)	9206 (13.2%)	
Living with spouse & children	6741 (88.2%)	37,688 (60.6%)	44,429 (63.6%)	
Living with children	414 (5.4%)	12,287 (19.8%)	12,701 (18.2%)	
Living with children & others	72 (0.9%)	1537 (2.5%)	1609 (2.3%)	

**Number of children**				
1 child	750 (9.8%)	5251 (8.4%)	6001 (8.6%)	<0.001
2 children	2758 (36.1%)	14,398 (23.2%)	17,156 (24.6%)	
3 children	1953 (25.6%)	15,547 (25.0%)	17,500 (25.1%)	
4 children	1055 (13.8%)	11,386 (18.3%)	12,441 (17.8%)	
5+ children	1122 (14.7%)	15,608 (25.1%)	16,730 (24.0%)	

## Data Availability

Data is publicly available for academic research purposes on request through the data request form. The data can be requested from https://www.iipsindia.ac.in/content/LASI-data.

## Footnotes

2 We refrain from using the terms “Average Treatment Effect” or “treatment and control groups” throughout the manuscript, except whenever we describe the methods, such as IPW, it is necessary to use these terms “treatment and control groups”.
